# Enhanced prefrontal functional–structural networks to support postural control deficits after traumatic brain injury in a pediatric population

**DOI:** 10.1162/NETN_a_00007

**Published:** 2017-06-01

**Authors:** Ibai Diez, David Drijkoningen, Sebastiano Stramaglia, Paolo Bonifazi, Daniele Marinazzo, Jolien Gooijers, Stephan P. Swinnen, Jesus M. Cortes

**Affiliations:** Biocruces Health Research Institute, Cruces University Hospital, Barakaldo, Spain.; KU Leuven, Movement Control and Neuroplasticity Research Group, Group Biomedical Sciences, Leuve, Belgium.; Dipartimento di Fisica, Universita degli Studi di Bari and INFN, Bari, Italy.; Basque Center for Applied Mathematics (BCAM), Bilbao, Spain.; Department of Data Analysis, Faculty of Psychological and Pedagogical Sciences, University of Ghent, Ghent, Belgium.; KU Leuven, Leuven Research Institute for Neuroscience & Disease (LIND), Leuven, Belgium.; Ikerbasque: The Basque Foundation for Science, Bilbao, Spain.; Department of Cell Biology and Histology, University of the Basque Country, Leioa, Spain.

**Keywords:** Traumatic brain injury, Prefrontal cortex, Network reorganization, Resting state, Functional networks, Structural networks, Brain hierarchical atlas

## Abstract

Traumatic brain injury (TBI) affects structural connectivity, triggering the reorganization of structural–functional circuits in a manner that remains poorly understood. We focus here on brain network reorganization in relation to postural control deficits after TBI. We enrolled young participants who had suffered moderate to severe TBI, comparing them to young, typically developing control participants. TBI patients (but not controls) recruited prefrontal regions to interact with two separated networks: (1) a subcortical network, including parts of the motor network, basal ganglia, cerebellum, hippocampus, amygdala, posterior cingulate gyrus, and precuneus; and (2) a task-positive network, involving regions of the dorsal attention system, together with dorsolateral and ventrolateral prefrontal regions. We also found that the increased prefrontal connectivity in TBI patients was correlated with some postural control indices, such as the amount of body sway, whereby patients with worse balance increased their connectivity in frontal regions more strongly. The increased prefrontal connectivity found in TBI patients may provide the structural scaffolding for stronger cognitive control of certain behavioral functions, consistent with the observations that various motor tasks are performed less automatically following TBI and that more cognitive control is associated with such actions.

## Author Summary

Using a new hierarchical atlas whose modules are relevant for both structure and function, we found increased structural and functional connectivity in prefrontal regions in TBI patients as compared to controls, in addition to a general pattern of overall decreased connectivity across the TBI brain. Although this increased prefrontal connectivity reflected interactions between brain areas when participants were at rest, the enhanced connectivity was found to be negatively correlated with active behavior such as postural control performance. Thus our findings, obtained when the brain was at rest, potentially reflect how TBI patients orchestrate task-related activations to support behavior in everyday life. In particular, our findings of enhanced connectivity in TBI might help these patients overcome deficits in cerebellar and subcortical connections, in addition to compensating for deficits when interacting with the task-positive network. Hence, it appears that greater cognitive control is exerted over certain actions in order to overcome deficits in their automatic processing.

## INTRODUCTION

Traumatic brain injury (TBI) involves brain tissue damage resulting from an external mechanical force, such as rapid head acceleration/deceleration or impact. On a neural level, TBI generally disrupts functional and structural large-scale brain networks (i.e., the networks of white matter tracts connecting different brain regions), while on a behavioral level, TBI often triggers various deficits, including cognitive impairments, motor problems, emotional sequelae, and so forth, that can persist for years postinjury (Caeyenberghs, Leemans, De Decker, et al., 2012; Ham & Sharp, 2012; D. H. Smith & Meaney, 2000). We focus here on deficits in balance control after TBI, which can last from months to several years after the traumatic impact in both adults (Guskiewicz, Riemann, Perrin, & Nashner, 1997; McCulloch, Buxton, Hackney, & Lowers, 2010) and children (Drijkoningen, Caeyenberghs, Vander Linden, et al., 2015; Drijkoningen, Leunissen, et al., 2015; Katz-Leurer, Rotem, Lewitus, Keren, & Meyer, 2008), which is psychosocially important because it increases the risk of falling, and thus affects the patient’s independence (McCulloch, Buxton, Hackney, & Lowers, 2010; Wade, Canning, Fowler, Felmingham, & Baguley, 1997).

Over the past decades, imaging techniques such as diffusion-weighted imaging (DWI) and functional magnetic resonance imaging (fMRI) have increased our understanding of the physiopathology of TBI. In particular, recent advances in MRI techniques have allowed for an analysis of the injured brain and for correlation of the damages there with behavior from a network perspective—that is, for exploring the structural and functional connectivity of neuronal networks in vivo (Barbey et al., 2015; Bonnelle et al., 2011; Caeyenberghs et al., 2014; Caeyenberghs, Leemans, De Decker, et al., 2012; Fagerholm, Hellyer, Scott, Leech, & Sharp, 2015; Ham & Sharp, 2012; Mäki-Marttunen, Diez, Cortes, Chialvo, & Villarreal, 2013; Sharp et al., 2011; Sharp, Scott, & Leech, 2014). For instance, diffusion-weighted results have revealed reduced structural connectivity and reduced network efficiency in TBI patients in relation to poorer cognitive functioning (Bonnelle et al., 2011; Caeyenberghs et al., 2014; Fagerholm et al., 2015; Kim et al., 2014) and poorer pediatric balance control (Caeyenberghs, Leemans, De Decker, et al., 2012). Moreover, with respect to resting-state functional connectivity—that is, looking at regional BOLD interactions when the brain is at rest—multiple studies have reported TBI-induced alterations (Bonnelle et al., 2012; Bonnelle et al., 2011; Hillary et al., 2011; Sharp et al., 2011; Sharp et al., 2014; Tarapore et al., 2013), even in cases of mild TBI (Mayer, Mannell, Ling, Gasparovic, & Yeo, 2011; Zhou et al., 2012). It has been shown, for instance, that TBI patients have increased functional connectivity within the default mode network (DMN), as compared to healthy controls (Hillary et al., 2011; Palacios et al., 2013; Sharp et al., 2011), which possibly acts as a compensatory mechanism for the loss of structural connections (i.e., axonal injury). Importantly, TBI-induced changes in resting-state functional connectivity seem to predict the development of attention impairments (Bonnelle et al., 2011). Finally, combining the information from structural and functional networks has resulted in better prediction of task-switching performance in TBI (Caeyenberghs, Leemans, Leunissen, Michiels, & Swinnen, 2013).

Although there is now sufficient evidence that TBI damages the large-scale and emerging properties of brain structural and functional networks, and that the degree of network impairment is correlated with behavioral and cognitive deficits (Drijkoningen, Caeyenberghs, Leunissen, et al., 2015; Palacios et al., 2013; Zhou et al., 2012), the precise pattern of structural–functional circuit reorganization after TBI is still poorly characterized. Here, we used a novel brain atlas Diez, Bonifazi, et al., 2015 to probe the working hypothesis that when structural networks are damaged and reorganized as a result of TBI, there is an associated reorganization of the corresponding functional networks, and vice versa, thus emphasizing the strong mutual relationship between brain structure and function (Damoiseaux & Greicius, 2009; Diez, Bonifazi, et al., 2015; Park & Friston, 2013). Moreover, in addition to previous work correlating white matter microstructural information with balance performance postinjury (Caeyenberghs et al., 2010; Caeyenberghs, Leemans, Heitger, et al., 2012; Drijkoningen, Caeyenberghs, Leunissen, et al., 2015), we aimed here to assess whether this structure–function network reorganization is in any manner related to critical behavior, such as the postural control deficits after TBI.

## MATERIALS AND METHODS

### Participants

The study included a total of 41 young subjects, 14 of whom had incurred a TBI (age: 13.14 ± 3.25 years; six males and eight females), along with 27 healthy control subjects who had developed normally (age: 15.04 ± 2.26 years; 12 males, 15 females). Group differences for age and sex yielded, respectively, *p* = 0.1306 (after a *t*-test) and *p* = 0.9226 (after a chi-squared test), so the TBI and control groups were age- and sex-matched. The TBI patients had suffered moderate to severe head injury, as defined by the Mayo classification system for injury severity. This system classifies patients according to the length of posttraumatic amnesia, loss of consciousness duration, lowest Glasgow Coma Scale score in the first 24 h, and MRI or computed tomography images, as assessed by a specialized clinical neurologist. Demographic data for all patients are given in Table 1. Independently of the specific lesions observed during the acute scan (column 2 in Table 2), at the time of the study all 14 TBI patients had diffuse axonal injury, and all of them had no severe focal lesions (column 3 in Table 2). The TBI patients’ mean age at the time of injury was 10 ± 3.45 years, and the average time interval between the injury and the present MRI was 3.5 ± 3.5 years. Exclusion criteria were based on preexisting developmental disorders, central neurological disorders, intellectual disabilities, and musculoskeletal disease. Additional exclusion criteria were having an abbreviated injury score above 2 for the upper or lower limbs, indicating seriously impaired limb function. The demographic and clinical descriptors of the TBI group are given in Tables 1 and 2.

**Table 1.** Demographic data of TBI patients.

**ID**	**Age**	**Gender (y)**	**Cause of Injury**	**Age at Injury (y)**	**Time Since Injury (y)**	**GCS/Coma Duration**
T01	8.6	M	TA	7.9	0.7	C: 5 days
T02	18.1	F	TA	15.6	2.5	C: 5 days
T03	9.3	F	TA	7.9	1.4	C: 2 weeks
T04	16.5	F	TA	7.2	9.3	N/A
T05	14.2	F	TA	7.7	6.5	N/A
T06	13.4	M	TA	12.5	0.8	N/A
T07	19.0	F	Fall	12.5	6.5	N/A
T08	15.6	M	TA	12.5	3.2	C: 10 days
T09	13.9	M	TA	13.5	0.3	GCS: 3
T10	8.5	F	TA	7.7	0.8	N/A
T11	11.4	M	Sports injury	9.8	1.5	N/A
T12	13.3	M	TA	12.1	1.2	N/A
T13	16.0	F	N/A	N/A	N/A	N/A
T14	13.8	F	Object Impact	3.0	10.8	N/A


M = male, F = female; TA = traffic accident, C = coma, GCS = Glasgow Coma Scale score, N/A = Information not available

**Table 2.** Clinical data of the TBI patients.

**ID**	**Acute MRI Scan Within 24 h After Injury Lesion Location/Pathology**	**MRI Scan Analyzed in This Study Lesion Location/Pathology**
T01	Subdural hematoma R FL/PL/TL; cortical contusion R FL/PL; DAI in R FL	Hemosiderin deposits: R semiovale center and CC
T02	Subdural hematoma/hemorrhagic contusion TL/FL; injuries R FL, thalamus, R cerebral peduncle, L mesencephalon; cortical and subcortical hemorrhagic areas in PL/TL	Small injuries surrounding drain trajectory in RH (superior frontal gyrus, head nucleus caudatus, crus anterius of internal capsule, thalamus, and pons)
T03	DAI in L TL/FL, R TL/FL/PL	Contusion: R anterior temporal pole and R orbitofrontal cortex; Injuries and atrophy in CC (body and splenium); Atrophy of R pons; Hemosiderin deposits in L cerebellar hemisphere, R nucleus lentiformis, L/R FL, L/R PL and R PL
T04	Epidural hematoma R FL/TL; shift midline	Injuries in R medial frontal gyrus.
T05	NA	Atrophy of the cerebellum; Injuries at the level of L FL, premotor cortex, L/R medial frontal gyrus, cingulate gyrus, orbitofrontal cortex (L R); Contusion anterior temporal pole (R L); Hemosiderin deposits in CC, L thalamus, striatum (R L)
T06	Hemorrhagic contusion L TL; brain edema	Hemosiderin deposits: several spread out over L/R PL, R cerebellum, L superior frontal gyrus. Hemociderosis as a remnant of subdural hemorrhage
T07	Subdural hematoma L FL/TL/PL	Hemosiderin deposits R cerebellar vermis
T08	DAI R TL, internal capsule, supra-orbital R FL, L FL WM (anterior corona radiata), L middle cerebellar peduncle	Atrophy cerebellum; Contusion R FL WM
T09	DAI FL, TL, L OL (hemorrhagic injury), cerebellum, CC, external capsule, R globus pallidus, L thalamus, R cerebral peduncle, R mesencephalon	Hemosiderin deposits: L FL, periventricular WM, body and genu CC, L thalamus, R external capsule, anterior TL (L R), L/R cerebellum; limited atrophy cerebellum
T10	NA	Enlarged fourth ventricle, atrophy of cerebellar vermis, contusion R cerebellar vermis, hypotrophy of middle cerebellar peduncle and L pons; contusion L TL; Hemosiderin deposits R FL, L TL, Vermis
T11	Contusion L FL/TL; Enlarged, asymmetric ventricle (temporal horn)	Hemosiderin deposit: splenium CC
T12	DAI in genu and splenium CC, L FL	Hemosiderin deposits L FL, genu CC
T13	NA	Mild atrophy in cerebellum and cerebrum, more pronounced atrophy in frontal cortices, enlarged ventricles; contusion L/R anterior temporal pole and L/R orbitofrontal cortex. Hemosiderin deposits in cerebellum, R FL
T14	Hemorrhagic contusion L FL, atrophy L FL	Contusion: L anterior middle frontal gyrus and L anterior superior frontal gyrus


WM = white matter, RH = right hemisphere, LH = left hemisphere, FL = frontal lobe, TL = temporal lobe, PL = parietal lobe, OL = occipital lobe, CC = corpus callosum; R = right, L = left.

The study was approved by the ethics committee for biomedical research at KU Leuven, and the patients were all recruited from several rehabilitation centers in Belgium (principal investigator, Stephan Swinnen). Written informed consent was obtained from either the participants themselves or the patients’ first-degree relatives, according to the Declaration of Helsinki.

### Balance Tests

Balance control was assessed using three protocols from the EquiTest System (NeuroCom International, Clackamas, Oregon).

#### Sensory organization test (SOT)

This test measures static postural control while subjects are standing as still as possible, barefoot, on a movable platform (forceplate) under four sensory conditions: (1) eyes open, fixed platform; (2) eyes closed, fixed platform; (3) eyes open with the platform tilting in response to body sway to prevent the ankles from bending (reduced somatosensory feedback); and (4) eyes closed, tilting platform. To familiarize the subject with the test and avoid any initial effect of surprise on the sensory manipulations, we included one practice trial for each condition prior to completing the actual measurements. After that, each condition was repeated three times in a randomized order. Each trial lasted 20 s. We used an established protocol that had been applied in earlier studies to assess balance control in both young and older healthy adults: calculating the center-of-pressure (COP) trajectory from the forceplate recordings (100 Hz) (Van Impe, Coxon, Goble, Doumas, Swinnen, 2012). A mean SOT balance score was acquired for each condition from the three trials, excluding trials in which the subject fell. We evaluated the behavioral outcome through the inverse path length (iPL) of the COP trajectory in order to acquire a SOT balance index in which higher scores reflected better balance control (and less body sway).

#### Limits-of-stability test (LOS)

This is a more dynamic test of balance control that involves goal-directed postural adjustments, in which subjects intentionally displace their center of gravity (COG) in different directions without stepping, falling, or lifting their heel or toes. At the beginning of each trial, the COG (provided by the Equitest forceplate) was positioned in the center, as indicated by a representation on a screen in front of the subject. On presentation of a visual cue and by leaning over in the right direction, the subject had to move the COG from the center toward one of the radial targets presented on the screen as quickly and accurately as possible. The following eight target directions were assessed: front, right front, right, right back, back, left back, left, and left front. After two practice trials, each direction was assessed once in a random order. The trial was interrupted and repeated if the subject fell or took a step, and that trial was not analyzed. Directional control (DC) was computed as the outcome measure reflecting dynamic balance control. Specifically, the DC (expressed as a percentage) was calculated as the difference between on-target (in the target direction) and off-target (extraneous) movement, divided by the amount of on-target movement, as follows: (amount of on-target movement – amount of off-target movement)/(amount of on-target movement) × 100%. Higher scores reflect better DC, and only a straight line toward the target would result in a score of 100%, with no off-target movements. Finally, to produce a single measure to be correlated with the imaging results, DC scores were averaged across the eight target directions for further analysis.

#### Rhythmic weight shift test (RWS)

Like the LOS, this is a dynamic test of balance control measuring the ability to move the COG rhythmically from right to left, or forward and backward, between two target positions. Each direction (backward–forward, left–right) was performed at three different speeds: slow (a pace of 3 s between each target), medium (a pace of 2 s), and fast (a pace of 1 s). Each combination of speed and direction (a total of six combinations) was performed in a separate trial of six movement repetitions that were preceded by four practice repetitions. The trial was interrupted and repeated if the subject fell or took a step. The DC was calculated as above (similar to LOS), and the DC scores were averaged across directions and velocities for further analysis.

In summary, postural control was evaluated by means of three different score indexes: one measuring static postural control (iPL-SOT), and two measuring dynamical postural control (DC-LOS and DC-RWS). The three indexes were used as behavioral outcome to correlate with the imaging results.

### Imaging

#### MRI acquisition

MRI scanning was performed on a Siemens 3-tesla Magnetom Trio MRI scanner with a 12-channel matrix head coil.

##### Anatomical data

A high-resolution T1 image was acquired with a 3D magnetization-prepared rapid-acquisition gradient echo (MPRAGE): repetition time [TR] = 2,300 ms, echo time [TE] = 2.98 ms, voxel size = 1 × 1 × 1.1 mm^3^, slice thickness = 1.1 mm, field of view [FOV] = 256 × 240 mm^2^, 160 contiguous sagittal slices covering the entire brain and brainstem.

##### Diffusion tensor imaging (DTI)

A DTI diffusion-weighted single-shot spin-echo echoplanar imaging sequence was acquired with the following parameters: TR = 8,000 ms, TE = 91 ms, voxel size = 2.2 × 2.2 × 2.2 mm^3^, slice thickness = 2.2 mm, FOV = 212 × 212 mm^2^, 60 contiguous sagittal slices covering the entire brain and brainstem. A diffusion gradient was applied along 64 noncollinear directions with a *b* value of 1,000 s/mm^2^. Additionally, one set of images was acquired with no diffusion weighting (*b* = 0 s/mm^2^).

##### Resting-state functional data

Resting-state fMRI time series were acquired over a 10-min session using the following parameters: 200 whole-brain gradient-echo echoplanar images with TR/TE = 3,000/30 ms, FOV = 230 × 230 mm^2^, voxel size = 2.5 × 2.5 × 3.1 mm^3^, 80 × 80 matrix, slice thickness = 2.8 mm, and 50 sagittal slices, interleaved in descending order.

#### MRI preprocessing

##### Diffusion tensor imaging

We applied DTI preprocessing similar to that in previous work (Alonso-Montes et al., 2015; Amor et al., 2015; Diez, Bonifazi, et al., 2015) using FSL (FMRIB Software Library, version 5.0) and the Diffusion Toolkit. First, an eddy current correction was applied in order to overcome the artifacts produced by variation in the direction of the gradient fields of the MR scanner, together with the artifacts produced by head movements. To ensure that group differences were not due to differences in motion, the average motion of each subject was used as a covariate of noninterest in the statistical analyses. In particular, the motion of the subject in the scanner was extracted from the transformation applied by the eddy current correction step from every volume to the reference volume (the first one, the *b* = 0 volume). Next, using the corrected data, a local fitting of the diffusion tensor was applied in order to compute the diffusion tensor model for each voxel. Next, a fiber assignment by continuous tracking algorithm was applied (Mori, Crain, Chacko, van Zijl, 1999). We then computed the transformation from the Montreal Neurological Institute (MNI) space to the individual-subject diffusion space and projected a high-resolution functional partition to the latter, composed of 2,514 regions of interest (ROIs), hereafter named simply *regions*, and generated after applying spatially constrained clustering to the functional data using in Craddock, James, Holtzheimer, Hu, and Mayberg (2012). This allowed for building 2,514 × 2,514 structural connectivity (SC) matrices, each per subject, by counting the number of white matter streamlines connecting all region pairs within the entire 2,514-region dataset. Thus, the element matrix (*i*, *j*) of SC was given by the streamline number between regions *i* and *j*. SC is a symmetric matrix, in which the connectivity from *i* to *j* is equal to that from *j* to *i*. Finally, we made the SC matrices binary for the analysis, considering only two possible values: 0 when no streamlines existed between *i* and *j*, and 1 when any nonzero number existed between the two regions *i* and *j*.

##### Resting-state fMRI

We applied resting-state fMRI preprocessing similar to that used in previous work (Alonso-Montes et al., 2015; Amor et al., 2015; Diez, Bonifazi, et al., 2015; Diez, Erramuzpe, et al., 2015; Mäki-Marttunen et al., 2013), by using FSL and AFNI (http://afni.nimh.nih.gov/afni/). First, slice-time correction was applied to the fMRI dataset; next, each volume was aligned to the middle volume to correct for head movement artifacts. All voxels were then spatially smoothed with a 6-mm full-width-at-half-maximum isotropic Gaussian kernel, and after intensity normalization, a bandpass filter was applied between 0.01 and 0.08 Hz (Cordes et al., 2001), followed by the removal of linear and quadratic trends. We next regressed out the movement time courses, the average cerebrospinal fluid (CSF) signal, the average white matter signal, and the average global signal. Finally, the functional data were spatially normalized to the MNI152 brain template, with a voxel size of 3 × 3 × 3 mm^3^. In addition to head motion correction, we performed scrubbing, by which means all time points with framewise displacement greater than 0.5 were interpolated by a cubic spline (Yan et al., 2013). Furthermore, to remove the effect of head movement in the group comparison analysis, we also used global frame displacement as a covariate of noninterest.

#### Clustering ROIs into modules by using a new hierarchical brain atlas

The initial 2,514 regions were grouped into modules using a recently published atlas (Diez, Bonifazi, et al., 2015), in which modules are regions that are functionally coherent (i.e., the dynamics of the voxels belonging to one module are very similar) and, at the same time, structurally wired (i.e., the voxels belonging to a given module are interconnected by white matter fibers). Some existing atlases are purely anatomical or structural (Desikan et al., 2006; Eickhoff et al., 2005; Lancaster et al., 2000; Tzourio-Mazoyer et al., 2002), and others are purely functional, such as those achieved after data-driven methods (Craddock et al., 2012). Although obtaining suitable brain partitions (or atlases) has been studied intensively (Craddock et al., 2013), to the best of our knowledge we were the first to propose a brain partition that accounts for modules that are relevant to both structure and function (Diez, Bonifazi, et al., 2015), which we now implemented in the present project.

Although full details are given in Diez, Bonifazi, et al. (2015), here we will briefly summarize the hierarchical clustering approach, which, when applied to a combination of functional and structural datasets, resulted in a hierarchical tree, or *dendrogram*, in which nodes were progressively merged together into *M* different modules following a nested hierarchy of “similarity” (which reflects both the correlations from the functional data and the numbers of white matter streamlines from the structural data). Thus, cutting the tree at a certain level led to pooling of the initial 2,514 ROIs into a finite number of modules, 1 ≤ *M* ≤ 2,514 (in principle, an arbitrary value for *M* could be obtained by varying the depth of the cut). Thus, to provide some examples, the highest dendrogram level, *M* = 1, corresponded to all 2,514 regions belonging to a single module, coincident with the entire brain, whereas the lowest level, *M* = 2,514, corresponded to 2,514 separate modules, all of them composed of a single region.

Diez, Bonifazi, et al. (2015) also showed that the hierarchical brain partition with *M* = 20 modules was optimal in terms of cross-modularity, an index simultaneously accounting for three features: (1) the modularity of the structural partition, (2) the modularity of the functional partition, and (3) similarity between the structural and functional modules. The MATLAB code to calculate the cross-modality index between structural and functional connectivity matrices can be downloaded from www.nitrc.org/projects/biocr_hcatlas/.

To compute cross-modularity, we first assessed modularity simply to account for the quality of the brain partition; that is, a partition with high modularity has modules highly isolated from each other—for instance, by maximizing the fraction of intramodule to intermodule connections with respect to randomization. In particular, we applied Newman’s algorithm to address modularity (Newman, 2004). In addition to modularity, cross-modularity made use of similarity between the structural and functional modules, which was approached by calculating Sorensen’s index, a normalized quantity equal to twice the number of common connections in the two modules, divided by the total number of connections in the two modules.

The entire hierarchical brain partition can be downloaded from www.nitrc.org/projects/biocr_hcatlas/.

### Statistical Analyses

#### Behavioral data

We compared our three balance control scores—iPL-SOT, DC-LOS, and DC-RWS—between the TBI and healthy control groups by using a two-tailed *t*-test.

#### Imaging data

##### Group differences in structural networks.

From the modules defined in the hierarchical atlas, structural networks (SN) were assessed by counting all the connections (i.e., streamlines) starting from one module and ending in a different one. Notice that modules can be defined at any level of the hierarchical tree. We then calculated the module’s connectivity degree (the total number of connections reaching a module, which, because SC is a symmetric matrix, coincides with the total number of connections leaving it).

Next, we applied a two-sample *t*-test using age and average head motion as covariates of noninterest, to search for significant differences (*p* 0.05). In particular, to test whether the means of two groups differed, we performed the hypothesis test using a general linear model, where *Y* contained the data and *X* the experimental design variables and confounds. Using the appropriate contrast (searching for mean group differences while removing the confound variables), we computed a two-sample unpaired *t*-test.

To assess the significance of the structural differences, we applied a permutation test by performing 1,000 random subject-label permutations. We then generated the probability distribution for these values, which constituted the null hypothesis, since all dependencies were removed by the shuffling procedure. All regions with *p* 0.05 were discarded.

As a final remark, although the original SC matrices of size 2,514 × 2,514 were binarized, at the module level we worked with weighted degrees for the group comparison analysis.

##### Group differences in resting-state brain dynamics within individual regions.

To determine group differences in the resting-state brain dynamics within each of the *M* = 20 modules, we first obtained the time series of the first principal component for each module, chosen as a representative for the entire module. Next, we compared four different descriptors extracted from these time series: variance (2nd standardized moment; quantifies fluctuation size), skewness (3rd standardized moment; identifies extreme brain dynamics in the resting state [Amor et al., 2015], which measures how much asymmetry a distribution has with respect to its mean), kurtosis (4th standardized moment; measures the long-tail effect on the data distribution), and the number of points resulting from the point process analysis (PPA; measured by counting the number of amplitude peaks in the BOLD signal [Tagliazucchi, Balenzuela, Fraiman, Chialvo, 2012], and in particular, counting the points with values greater than the mean value of the time series plus 1 *SD*). These descriptors were subjected to a two-sample *t*-test with age and head motion as covariates in order to evaluate the differences between the TBI and control participants (*p* 0.05).

##### Group differences in functional networks.

Motivated by an earlier study (S. M. Smith et al., 2009), functional networks (FN) were assessed by quantifying the interactions between each of the *M* = 20 modules and the rest of the brain (Figure S1). First, within each of the *M* = 20 modules, we applied a principal component analysis (PCA) so as to reduce the dimensionality of the data, resulting in 20 components for each of the *M* = 20 modules. Next we applied an independent component analysis (ICA), to obtain *C* = 20 independent time-series components associated with each of the *M* = 20 modules. Finally, we applied a general linear model, to quantify the contribution of each brain voxel to each component (i.e., component’s spatial map). Then we clustered all of the spatial maps by applying the *k*-means clustering algorithm, using the spatial correlation between observations as the similarity measure (Bishop, 2006); thus, two maps belonged to the same cluster if they showed high spatial correlation. After applying *k*-means, the 820 observations per module (41 subjects with *C* = 20 independent components each) were grouped into five clusters, which we named the five *most representative clusters* (MRCs). Here, the number 5 was chosen after careful inspection, to guarantee good discrimination between the different clusters. The *k*-means analysis, in addition to returning the five MRCs, also provided a label for each of the 820 observations—1, 2, 3, 4, or 5—indicating the MRC to which the observation belonged.

As a result of PCA + ICA, we obtained 20 spatial maps per module and subject. Each spatial map—that is, an observation—was assigned to one of the five MRCs, and this was done for each module. We took all the spatial maps, per MRC and module, and performed a *t*-test comparing the TBI group and the healthy controls.

To correct for multiple comparisons due to a voxel-by-voxel analysis, a statistically significant cluster-level family-wise error (FWE) was applied. In particular, a Monte Carlo simulation (3dClustSim; AFNI, http://afni.nimh.nih.gov) was performed with 10,000 iterations to estimate the probability of false-positive clusters with *p* 0.05, corrected with FWE. We used the new version of the 3dClustSim program (included in AFNI) software, which corrects for a bug detected by Eklund, Nichols, Knutsson (2016). After correcting for multiple comparisons, three classes of activity maps for each region were calculated: (1) the average FN in control participants (corresponding to the contrast [1 0 0 0], where the last two zeros correspond to the movement and age variables); (2) the average FN in TBI patients (contrast [0 1 0 0]); and (3) the differences between the average FNs of control and TBI participants (by applying the different contrasts [1 –1 0 0] and [–1 1 0 0], we calculated, respectively, control TBI connectivity and TBI control connectivity).

Note that all of the TBI patients in the MRI session used for this study had diffuse axonal injury, with no severe focal lesions or regional atrophy, which justified pooling all of the TBI patients into the same group to be compared with a group of healthy controls.

#### Relationship between the behavioral and imaging data

We used a general linear model that included the age and average frame displacement as covariates of noninterest to estimate the relationship between postural control and the variance/kurtosis/skewness/PPA in every voxel. Next, we used a *t*-test to assess the association between the postural control scores (iPL-SOT, DC-LOS, and DC-RWS) and the different fMRI measures, using 3dClustSim with a cluster-based FWE multiple-comparison correction. Within this region, we used the mask of TBI control structural connectivity and correlated the variance/kurtosis/skewness/PPA of voxels within this region with the three behavioral scores. To assess the association between the postural control variable and the fMRI variables, we took the region that survived the multiple comparison correction and plotted the correlation of the *corrected* variance (i.e., the variance after removing the effects of age and head motion) and the postural control variable. We performed both Pearson and Spearman correlational analyses, since the latter are less affected by the presence of outliers.

## RESULTS

### TBI-Induced Alterations in Postural Control Performance

Alterations in postural control performance were measured through three different tests (i.e., SOT, LOS, RWS). First, the SOT showed that TBI patients had a smaller inverse path length in the COP trajectory than did control participants (iPL-SOT: control, 106.72 ± 8.41; TBI, 88.63 ± 28.80; *p* = 0.0041; *t* = 3.054), which reflects that TBI patients had worse balance (more body sway) than the controls. Similarly, the RWS test showed that TBI patients performed more poorly than controls (DC-RWS: control, 83.77 ± 7.01; TBI, 77.11 ± 9.82; *p* = 0.0174; *t* = 2.4868), confirming poorer dynamic balance among TBI patients. In contrast, the LOS test did not show significant group differences as measured by the dynamic control index (DC-LOS: control, 83.80 ± 6.75; TBI, 83.46 ± 5.89; *p* = 0.8731; *t* = 0.1608).

### TBI-Induced Alterations in Structural Networks

**Figure 1.**
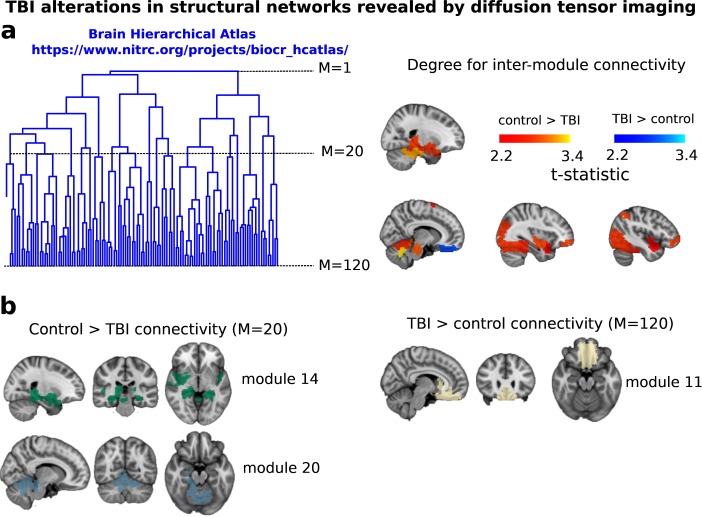
TBI-induced alterations to structural networks revealed by diffusion tensor imaging. (a) Hierarchical tree or dendrogram defining a hierarchal brain partition (Diez, Bonifazi, et al., 2015) in which three different levels of the tree have been emphasized: *M* = 1, where all brain regions belong to a single module; *M* = 20, the optimal brain partition (see Materials and Methods); and *M* = 120, the level at which structural connectivity was higher in TBI patients than in controls. Group differences were calculated on module degree maps derived from the intermodule connectivity matrix and after a two-sample *t*-test with age and head motion as covariates of noninterest (*p* 0.05). Multiple comparison corrections were achieved by applying subject-label permutations, thereby building the a null-hypothesis distribution, since all correlations were removed by this shuffling. Greater connectivity in controls than in TBI patients (red scale) was found at *M* = 20, and at *M* = 120, TBI control connectivity was also found (blue scale). Brain maps represent values of the *t*-statistic. (b)****At *M* = 20 (left graph), significant control TBI connectivity was evident in Module 14 (including parts of the hippocampus and parahippocampal gyrus, amygdala, putamen, insula, ventral diencephalon, temporal gyrus, and temporal pole) and Module 20 (including parts of the cerebellum and parahippocampal gyrus). At *M* = 120 (right graph), TBI control connectivity was found within Module 11, including parts of the rectus and superior and inferior frontal orbital gyri. The module colors are just indicative and coincide with the colors used in Diez, Bonifazi, et al. (2015), where we first published the hierarchical brain atlas.

Alterations in structural networks were assessed by calculating the connectivity degree for each module in the hierarchical atlas from the intermodule connectivity matrix and performing a group comparison (after correcting for multiple comparisons by performing random subject-label permutations). At the level of *M* = 20 modules (Table 3), control participants showed a greater number of connections reaching another module than did TBI patients (Figure 1). This suggests that a global decrease in connectivity is associated with TBI. More specifically, significant differences in connectivity degree were evident within Module 14 (*p* = 0.01, *t* = 2.64), which included parts of the hippocampus and parahippocampal gyrus, amygdala, putamen, insula, ventral diencephalon, temporal gyrus, and temporal pole, and Module 20 (*p* = 0.003, *t* = 3.13), which included parts of the cerebellum and parahippocampal gyrus. See Table 4 for full statistical details.

**Table 3.** Anatomical description of the *M* = 20 modules (along with volumes) in the hierarchical atlas published recently (Diez, Bonifazi, et al., 2015) and available to download at www.nitrc.org/projects/biocr_hcatlas/.

**Module (Volume)**	**Anatomical Description**
**Module** 1 (7.26 cm^3^)	**Posterior cingulate:** posterior area of the cingulate gyrus or callosal convolution. Located above the corpus callosum, it goes from the frontal lobe back to the temporal uncus and up to the splenium. It belongs to the default mode network.

Module 2 (104.36 cm^3^)	**Putamen:** a round structure located at the base of the telencephalon. It is also one of the basal ganglia structures.
	**Anterior cingulate:** anterior frontal region of the cingulate gyrus, initiated above the rostrum of the corpus callosum.
	**Rostral pars of middle frontal gyrus:** anterior inferior end of the middle frontal gyrus.
	**Superior parietal gyrus:** parietal gyrus located posterior to the postcentral gyrus.
	**Supramarginal gyrus**: region in the parietal lobe encircling the posterior extreme of the Sylvian fissure.
	**Insula:** triangular area of cerebral cortex forming the medial wall of the Sylvian fissure.
	**Inferior parietal gyrus:** parietal gyrus located behind the postcentral gyrus and below the superior parietal gyrus.
	**Precentral gyrus:** frontal gyrus that defines the anterior boundary of the fissure of Rolando with a mainly motor function.
	**Superior frontal gyrus:** antero-superior parasagittal frontal gyrus, located anterior to the precentral gyrus.

Module 3 (221.18 cm^3^)	**Paracentral lobule:** medial gyrus that connects the pre- and postcentral gyrus.
	**Precentral gyrus** (cf. Module 2)
	**Postcentral gyrus:** Parietal gyrus located between the fissure of Rolando and the postcentral sulcus, which has a mainly sensory function.
	**Precuneus:** square brain lobule located before the parieto-occipital sulcus and behind the paracentral lobule at the medial surface of the brain hemisphere.
	**Superior frontal gyrus** (cf. Module 2).
	**Superior parietal gyrus** (cf. Module 2)
	**Superior temporal gyrus:** temporal gyrus at the lateral surface of the temporal lobe. It is located below the Sylvian fissure and above the superior temporal sulcus. It belongs to the temporal neocortex.
	**Supramarginal gyrus** (cf. Module 2).
	**Insula** (cf. Module 2)

Module 4 (91.48 cm^3^)	**Cuneus:** occipital gyrus between the parieto-occipital sulcus and the calcarine sulcus at the medial surface of the occipital lobe.
	**Lateral occipital sulcus:** external lateral surface of the occipital lobe close to the occipital lobe, dividing the external occipital gyrus.
	**Lingual gyrus:** occipital extension of the parahippocampal gyrus at the medial surface of the occipital lobe.
	**Pericalcarine cortex:** occipital area encircling the calcarine sulcus with a function associated to visual perception.
	**Precuneus** (cf. Module 3)

Module 5 (37.02 cm^3^)	**Medial frontal gyrus:** frontal gyrus at the lateral surface below the superior frontal gyrus.
	**Precentral gyrus** (cf. Module 2)
	**Rostral pars of middle frontal gyrus** (cf. Module 2)

Module 6 (159.33 cm^3^)	**Cerebellum:** posterior part of the rombencephalon made up of the two hemispheres and the central vermis. It is located below the occipital lobe.
	**Fusiform gyrus:** temporal gyrus in the inferior surface between the inferior temporal gyrus and the parahippocampal gyrus. It has two areas, the medial occipito-temporal gyrus and the lateral occipito-temporal gyrus.
	**Inferior temporal gyrus:** inferior gyrus located in the lateral surface of the temporal lobe, below the inferior temporal sulcus.
	**Lateral occipital sulcus** (cf. Module 4)
	**Superior parietal gyrus** (cf. Module 2)

Module 7 (22.30 cm^3^)	**Thalamus:** middle symmetrical structure of the diencephalon with multiple afferent and efferent connections, situated around the third ventricle.
	**Caudate nucleus** (symmetrical structure): one of the basic structures of the basal ganglia belonging to the corpus striatum. It is located at the lateral surface of the lateral ventricles surrounding the thalamus.
	**Putamen** (cf. Module 2)
	**Pallidum:** symmetrical structure within the basal ganglia. Medial diencephalic region of the lenticular nucleus.
	**Accumbens nucleus:** symmetrical structure located in the ventral region of the corpus striatum, therefore belonging to the basal ganglia.

Module 8 (3.29 cm^3^)	**Caudate nucleus** (cf. Module 7)
	**Putamen** (cf. Module 2)

Module 9 (163.67 cm^3^)	**Cerebellum** (cf. Module 6)
	**Caudal middle frontal:** frontal gyrus on the lateral surface located below and lateral to the superior frontal gyrus. This region refers to its most caudal part.
	**Cingulate isthmus:** intersection narrowing between the cingulate and the hippocampal gyrus. It is located behind and below the splenium of the corpus callosum.
	**Posterior cingulate** (cf. Module 1)
	**Precuneus** (cf. Module 3)
	**Inferior parietal gyrus** (cf. Module 2)
	**Rostral pars of middle frontal gyrus** (cf. Module 2)
	**Superior frontal gyrus** (cf. Module 2)

Module 10 (103.55 cm^3^)	**Anterior cingulate** (cf. Module 2)
	**Inferior parietal gyrus** (cf. Module 2)
	**Orbital gyrus:** frontobasal gyrus lateral located to the straight gyrus.
	**Pars opercularis:** opercular part of the inferior frontal gyrus.
	**Pars orbitalis:** orbital part of the inferior frontal gyrus.
	**Pars triangularis:** inferior part of the inferior frontal gyrus.
	**Anterior cingulate** (cf. Module 2)
	**Rostral pars of middle frontal gyrus** (cf. Module 2)
	**Superior frontal gyrus** (cf. Module 2)

Module 11 (31.08 cm^3^)	**Caudate nucleus** (cf. Module 7)
	**Accumbens nucleus** (cf. Module 7)
	**Lateral frontal orbital gyrus:** external orbital gyrus, located frontobasal and lateral to the medial orbitofrontal gyrus.
	**Orbital gyrus** (cf. Module 10)
	**Anterior cingulate** (cf. Module 10)

Module 12 (33.24 cm^3^)	**Inferior parietal gyrus** (cf. Module 2)
	**Inferior temporal gyrus** (cf. Module 6)
	**Lateral frontal orbital gyrus** (cf. Module 11)
	**Pars orbitalis** (cf. Module 10)
	**Pars triangularis** (cf. Module 10)
	**Rostral pars of middle frontal gyrus** (cf. Module 2)
	**Superior frontal gyrus** (cf. Module 2)
	**Caudate nucleus and anterior cingulate** (cf. Modules 7 and 2)

Module 13 (24.46 cm^3^)	**Middle frontal gyrus:** caudal part of the middle frontal gyrus.
	**Pars opercularis** (cf. Module 10)
	**Precentral gyrus** (cf. Module 2)
	**Superior frontal gyrus** (cf. Module 2)

Module 14 (92.75 cm^3^)	**Thalamus** (cf. Module 7)
	**Hippocampus:** symmetrical grey matter structure, located in the medial-temporal region, at the base of the temporal horn.
	**Amygdala:** grey nuclei located in the temporal uncus, above the temporal ventricular horn. It belongs to the rhinencephalon.
	**Putamen** (cf. Module 2)
	**Ventral diencephalon:** multiple structures containing the hypothalamus, mammillary tubercle, subthalamic nucleus, substantia nigra, red nucleus, geniculate body, optic tract and cerebral peduncles.
	**Banks of the superior temporal sulcus:** Temporal lobe structure between the superior temporal gyrus and the middle temporal gyrus.
	**Parahippocampal gyrus:** convolution located below the hippocampal sulcus in the temporal mesial region.
	**Superior temporal gyrus** (cf. Module 3)
	**Insula** (cf. Module 2)
	**Middle temporal gyrus:** gyrus located on the lateral surface of the temporal lobe between the inferior and superior temporal sulcus.
	**Temporal pole:** anterior end of the temporal lobe.

Module 15 (42.96 cm^3^)	**Thalamus** (cf. Module 7)
	**Putamen** (cf. Module 2)
	**Pallidum** (cf. Module 7)
	**Brainstem:** it consists of three parts, the myelencephalon, pons (metencephalon) and midbrain (mesencephalon). It is the main communication route between the brain, spinal cord, and peripheral nerves.
	**Hippocampus** (cf. region14)
	**Amygdala** (cf. Module 14)
	**Accumbens nucleus** (cf. Module 7)
	**Ventral diencephalon** (cf. Module 14)
	**Orbital gyrus** (cf. Module 10)
	**Insula** (cf. Module 2)

Module 16 (65.58 cm^3^)	**Cerebellum** (cf. Module 6)
	**Banks of the superior temporal sulcus** (cf. Module 14)
	**Inferior parietal gyrus** (cf. Module 2)
	**Cingulate isthmus** (cf. Module 9)
	**Middle temporal gyrus** (cf. Module 14)
	**Precuneus** (cf. Module 3)
	**Superior temporal gyrus** (cf. Module 3)

Module 17 (5.29 cm^3^)	**Banks of the superior temporal sulcus** (cf. Module 14)
	**Middle temporal gyrus** (cf. Module 14)

Module 18 (74.39 cm^3^)	**Hippocampus** (cf. Module 14)
	**Amygdala** (cf. Module 14)
	**Entorhinal cortex**: area in the medial-temporal lobe located between the hippocampus and temporal neocortex.
	**Fusiform gyrus** (cf. Module 6)
	**Inferior temporal gyrus** (cf. Module 6)
	**Middle temporal gyrus** (cf. Module 14)
	**Parahippocampal gyrus** (cf. Module 14)
	**Temporal pole** (cf. Module 14)

Module 19 (28.54 cm^3^)	**Cerebellum** (cf. Module 6)
	**Brainstem** (cf. Module 15)

Module 20 (34.91 cm^3^)	**Cerebellum** (cf. Module 6)
	**Parahippocampal gyrus** (cf. Module 14)

**Table 4.** TBI versus control differences with respect to structural networks revealed by diffusion tensor imaging (cf. Figure 1).

**At the level of *M*** = **20 modules**					
**Module**		***t*-Statistic**	***p*-Value**	**Effect Size (Hedges)**	**Confidence Intervals**
14		2.6351	0.0120	0.8511	0.1539, 0.2990
20		3.1276	0.0033	1.0101	1.5152, 1.6821
**At the level of *M*** = **120 modules**					
**Module 120**	**Module 20**	***t*-Statistic**	***p*-Value**	**Effect Size (Hedges)**	**Confidence Intervals**
1	11	–2.7511	0.0090	–0.8885	–1.5544, –0.1883
25	12	2.6874	0.0105	0.8679	0.1694, 1.5329
45	13	2.2003	0.0338	0.7106	0.0244, 1.3694
70	6	2.6289	0.0122	0.8491	0.1521, 1.5131
72	6	2.4432	0.0192	0.7891	0.0969, 1.4507
79	14	2.2327	0.0314	0.7211	0.0341, 1.3802
84	14	2.7039	0.0101	0.8733	0.1743, 1.5384
85	14	2.4728	0.0179	0.7986	0.1057, 1.4606
87	14	2.8865	0.0063	0.9323	0.2282, 1.6002
88	14	3.4186	0.0015	1.1041	0.3840, 1.7815
106	18	2.4281	0.0199	0.7842	0.0924, 1.4456
114	19	2.7931	0.0080	0.9021	0.2007, 1.5686
117	20	3.3141	0.0020	1.0703	0.3535, 1.7457
118	20	2.5526	0.0147	0.8244	0.1295, 1.4874
119	20	2.2096	0.0331	0.7136	0.0272, 1.3725

At the level of *M* = 20 in the hierarchical tree, the intermodule connectivity degree was higher in controls than in patients, indicating that one must go down the hierarchical tree to find a representation with a higher spatial scale (i.e., the number *M* of modules increases lower down on the tree), where TBI connectivity might be higher than control connectivity. Proceeding in this way, at the level of *M* = 120 modules we found higher connectivity values for TBI patients than for controls within Module 11 of the hierarchical atlas (*p* = 0.009, *t* = 2.75). This module includes parts of the caudate nucleus, nucleus accumbens, lateral frontal orbital gyrus, orbital gyrus, and anterior cingulate gyrus. Thus, whereas at the level of *M* = 20 TBI reduced participants’ connectivity relative to controls, at the level of *M* = 120 modules (i.e., at a higher spatial scale), prefrontal regions showed an increase in connectivity for TBI as compared to controls.

### TBI-Induced Alterations in Resting-State Brain Dynamics Within Individual Modules

**Figure 2.**
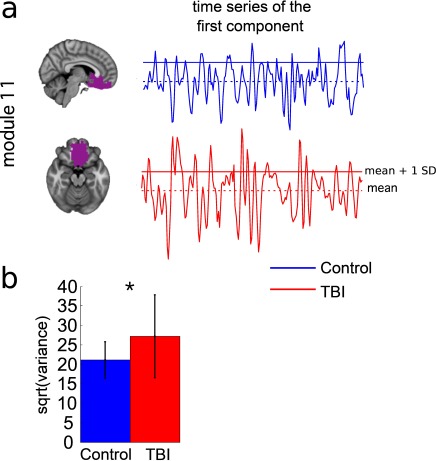
TBI-induced alterations to brain dynamics within individual modules revealed by resting-state fMRI. For each of the *M* = 20 modules in the hierarchical atlas, we extracted the time series of the first principal component and calculated four different descriptors: the variance, skewness, kurtosis, and number of points after the point process analysis (PPA; Materials and Methods). (a) Only Module 11 showed differences between the TBI and control groups with respect to the variance of the time series of the first principal component. The dashed lines represent the mean value of the time series, and the solid lines represent the threshold used for the PPA, here equal to the mean + 1 *SD*. (b) For Module 11, the variance of the first component (plotted here as its square root—i.e., the standard deviation) differed between TBI and control subjects. In particular, the fact that the variance was higher in TBI (red) than in controls (blue) showed compensation rather than a deficit. The color of Module 11 (magenta) is just indicative and coincides with the color used in Diez, Bonifazi, et al. (2015), where we first published the hierarchical brain atlas.

Alterations in the resting-state brain dynamics within each of the *M* = 20 modules were assessed by calculating differences in the time series of the first principal component extracted from each module (Figure 2). The explained data variance across modules varied from 28% to 54%, with a mean value of 38%. In particular, differences emerged with respect to the second moment (i.e., *variance*), the third moment (*skewness*), the fourth moment (*kurtosis*), and the number of time-series points that had a value above the mean value of the time series plus 1, times the standard deviation. After repeating the same procedure for all *M* = 20 modules of the hierarchical atlas, significant differences emerged only within Module 11 (*p* = 0.01, *t* = –2.55; explaining 48.55% of the data variance of the first principal component), revealing that the variance of brain dynamics was higher in the TBI than in the control group. Full statistical details are given in Table 5.

**Table 5.** TBI versus control differences with respect to brain dynamics within individual modules revealed by resting-state fMRI (cf. Figure 2).

**Module**	***t*-Statistic**	***p*-Value**	**Effect Size (Hedges)**	**Confidence Intervals**
11 (variance)	–2.5512	0.0148	–0.8240	–1.4870, –0.2000

### TBI-Induced Alterations in Functional Networks

Functional networks were addressed by quantifying the interaction of each of the *M* = 20 modules with the rest of the brain. Within each module, we first obtained *C* = 20 components (after PCA followed by ICA), and next we performed spatial regression of the *C* = 20 components to all the brain voxels, in this way obtaining *C* = 20 spatial maps for each of the modules. We grouped all 820 of the observations (41 subjects, each with *C* = 20 independent components) per each module into the five MRCs.

**Figure 3.**
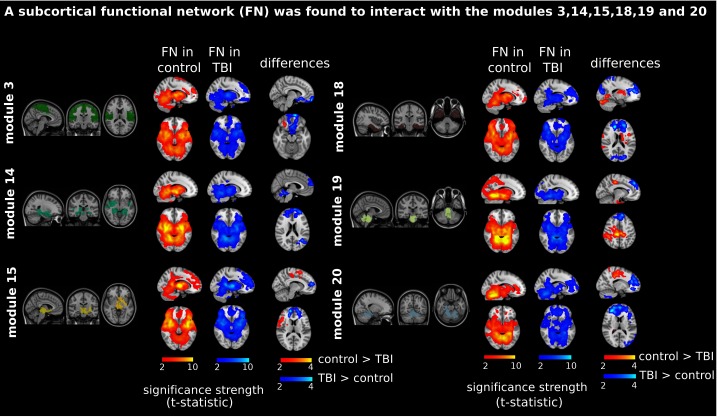
Prefrontal recruitment into a subcortical functional network (FN). Significant brain maps show different contrasts: column 1, “FN in control,” with a red bar and corresponding to the contrast [1 0 0 0] (see Materials and Methods); column 2, “FN in TBI,” with a blue bar and corresponding to the contrast [0 1 0 0]; and column 3, “differences,” corresponding to two contrasts, [1 –1 0 0] and [–1 1 0 0], represented in red (control TBI activation) and blue (TBI control activation), respectively. TBI patients (but not control participants) recruited the prefrontal part of the brain when interacting with a subcortical network (colored in blue at the “differences” column). In all cases, the bar scale represents the strength of significance, measured by the *t*-statistic values. Contrasts [1 0 0 0] and [0 1 0 0] define the “subcortical network” (which corresponds to one most representative cluster including parts of the cerebellum, basal ganglia, thalamus, amygdala, and temporal poles). This network resulted from the interactions of Module 3 (including parts of the sensory–motor and auditory networks), Modules 14 and 15 (including parts of the thalamus, hippocampus, amygdala, putamen, ventral diencephalon, and insula), Module 18 (including parts of the hippocampus and entorhinal cortex, fusiform gyrus, inferior and middle temporal gyrus, and parahippocampal gyrus), Module 19 (including parts of the cerebellum and brainstem), and Module 20 (including parts of the cerebellum and parahippocampal gyrus). The module colors are just indicative and coincide with the colors used in Diez, Bonifazi, et al. (2015), where we first published the hierarchical brain atlas.

After this procedure, it was possible to obtain the same MRC from different modules. In particular, Figure 3 shows the results associated with one of the MRCs, obtained from the following modules: Module 3 (including parts of the sensory–motor and auditory networks), Modules 14 and 15 (including parts of the thalamus, hippocampus, amygdala, putamen, ventral diencephalon, and insula), Module 18 (including parts of the hippocampus and entorhinal cortex, fusiform gyrus, inferior and middle temporal gyrus, and parahippocampal gyrus), Module 19 (including parts of the cerebellum and brainstem), and Module 20 (including parts of the cerebellum and parahippocampal gyrus).

The anatomical representation of this MRC (obtained from Modules 3, 14, 15, 18, 19, and 20) revealed a *subcortical network* (see Figure 3, “FN in control” and “FN in TBI” columns), consisting of part of the motor network, basal ganglia, cerebellum, thalamus, parahippocampus, hippocampus, precuneus, amygdala, insula, caudate nucleus, putamen, and pallidum. Interestingly, the TBI control connectivity comparison (obtained with the contrast [–1 1 0 0]) revealed one cluster in the frontal lobe (Figure 3, column “differences,” colored in blue), a region including part of the middle frontal and superior orbital gyri, rectus, olfactory lobe, frontal medial orbital, precuneus, and anterior cingulate gyrus. In other words, the subcortical network illustrated in Figure 3 with the labels “FN in control” and “FN in TBI” recruited the prefrontal brain in TBI patients but not in control participants.

**Figure 4.**
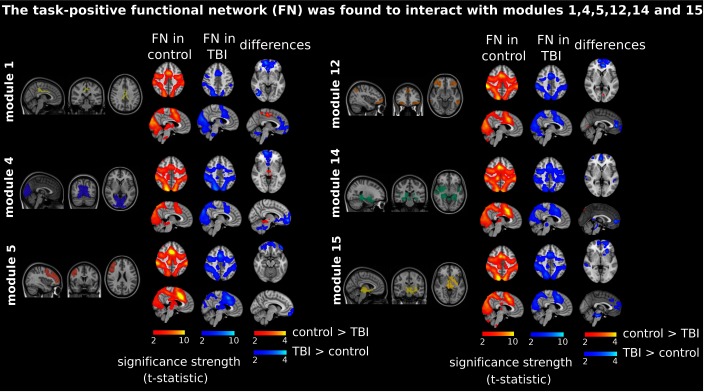
Prefrontal recruitment into the task-positive functional network (FN). These are depicted as in Figure 3, but the most representative cluster now resembles the task-positive network (see the labels “FN in control” and “FN in TBI”), which is now resulting from Module 1 (posterior cingulate cortex), Module 4 (medial visual cortex), Module 5 (medial frontal gyrus), Module 12 (inferior parietal and temporal gyrus, lateral frontal orbital gyrus, rostral pars of middle frontal gyrus, and pars orbitalis and triangularis), and Modules 14 and 15 (subcortical structures). Similar to what is shown in Figure 3, now TBI patients recruited the prefrontal part of the brain in interaction with the task-positive network (colored in blue in the “differences” column).

A different MRC resembled the *task-positive network* (Figure 4, “FN in control” and “FN in TBI” columns). In particular, this MRC consisted of parts of the cerebellum, lingual gyrus, fusiform gyrus, inferior occipital gyrus, calcarine sulcus, cuneus, precuneus, superior temporal pole, superior motor area, and insula. The MRC resulted from the functional interactions between Module 1 (including part of the posterior cingulate gyrus), Module 4 (the medial visual network), Module 5 (including parts of the medial frontal and precentral gyri and rostral pars of the middle frontal gyrus), Module 12 (including parts of the inferior parietal gyrus, inferior temporal gyrus, lateral frontal orbital gyrus, pars orbitalis, pars triangularis, rostral pars of middle frontal gyrus, superior frontal gyrus, caudate nucleus, and anterior cingulate gyrus), and Modules 14 and 15 (including parts of the thalamus, hippocampus, amygdala, putamen, ventral diencephalon, and insula).

Although both the control and TBI groups revealed similar task-positive networks (see Figure 4), the TBI connectivity control connectivity contrast (Figure 4, colored blue in the “differences” column) revealed a network in the frontal brain—more specifically, a network including parts of the frontal medial orbital, anterior cingulate, precuneus, superior frontal, and angular gyrus. Thus, like the subcortical network represented in Figure 3, the task-positive network recruited the prefrontal cortex in TBI patients but not in control participants.

### Brain Regions Showing Increased Connectivity in TBI for Both Functional and Structural Networks

**Figure 5.**
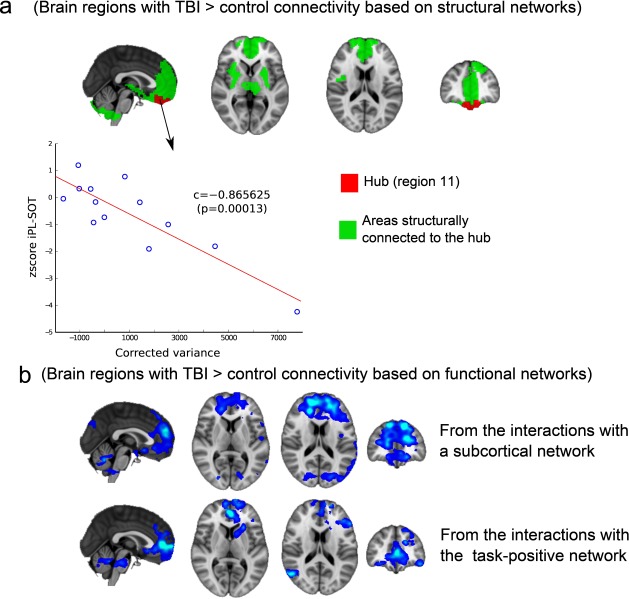
Common regions where TBI control connectivity resulted from both the structural and functional network analyses. (a) Structural network compensation. TBI control structural connectivity occurred within a subnetwork consisting of a hub (colored in red) connected to other regions (colored in green); the hub includes the orbitofrontal and rectus regions and belongs to Module 11 in the *M* = 20 hierarchical atlas. All of the following regions are connected to the hub: frontal superior regions, anterior cingulate gyrus, thalamus, striatum, insula, amygdala, hippocampus and parahippocampus, olfactory cortex, and cerebellum. The corrected variance of the first principal component of Module 11 was also correlated with postural control measures; here this is represented by the iPL-SOT score. (b) Functional network compensation. TBI control functional connectivity (blue) occurred when interacting with subcortical structures (including the superior frontal gyrus, superior medial frontal and middle frontal gyri, and anterior cingulate) and the task-positive network (including the anterior cingulate, the medial frontal and middle orbital gyri, the superior frontal medial gyrus, and the rectus). For both situations, the spatial maps represent the functional results of averaging all of the spatial maps with contrast [–1 1 0 0] in Figure 3 (subcortical network) and Figure 4 (task-positive network). Thus, TBI patients (but not control participants) incorporated prefrontal parts of the brain into both the subcortical and task-positive networks.

Because we observed increased connectivity in TBI patients relative to controls for both functional and structural networks, we decided to take a closer look at these overlapping findings by superimposing the regions (Figure 5). With regard to the analysis performed for the structural networks, a higher degree of connectivity in TBI patients was found in a small subnetwork, composed of a hub (Figure 5a, plotted in red) connected to other regions (Figure 5a, areas in green). The region’s hub belonged to Module 11 in the hierarchical atlas and connected to superior frontal regions, anterior cingulate, thalamus, striatum, insula, amygdala, hippocampus and parahippocampus, olfactory lobe, and cerebellum.

With regard to the analysis performed for the functional networks (Figure 5b), two regions showed increased connectivity in TBI relative to controls: one region interacting with a subcortical network (including superior frontal gyrus, superior medial frontal gyrus and middle frontal gyrus, and anterior cingulate) and another region interacting with the task-positive network (including anterior cingulate gyrus, frontal medial gyrus, middle orbital gyrus, superior frontal medial gyrus, and rectus).

By looking at both Figures 5a and 5b, we can see an overlap between the maps of increased structural connectivity (Figure 5a) and the maps corresponding to increased functional connectivity (Figure 5b), which occurred for both the subcortical and task-positive networks.

### Relation Between Postural Control and Prefrontal Dynamics at Rest

We found within Module 11 of the hierarchical atlas increased connectivity in both structural and functional networks for TBI patients in comparison to control participants. Correlational analyses revealed that the prefrontal activation dynamics of Module 11 at rest (represented by the *corrected* variance of the voxel fMRI dynamics) was correlated with the inverse path-length score of the static SOT (iPL-SOT), giving a Pearson correlation of *r* = –0.86 (*p* = 0.00013) and a Spearman correlation of *s* = –0.78 (*p* = 0.0026). These results suggest that better balance performance is associated with decreased dynamic activation in Module 11. Neither DC-RWS nor DC-LOS was significantly correlated with any of the fMRI measures within Module 11.

## DISCUSSION

Here we have provided the first evidence that TBI-induced alterations in functional and structural networks show overlapping results. With respect to both neuronal networks, TBI patients demonstrated increased prefrontal connectivity, relative to controls. Moreover, these TBI-induced network alterations were associated with changes in balance performance.

### TBI-Induced Alterations in Structural Networks

In agreement with previous studies (Gentry, Godersky, Thompson, 1988; Hulkower, Poliak, Rosenbaum, Zimmerman, Lipton, 2013; Zappalà, Thiebaut de Schotten, Eslinger, 2012), we found that TBI patients showed reduced structural connectivity (i.e., a smaller degree of connectivity) for most brain areas, as compared to healthy participants. More precisely, we found a strong decrease in connectivity degree in motor areas, brainstem, cingulate gyrus, cerebellum, and the temporal poles, areas that are typically associated with the performance of motor skills and balance control. Indeed, decreased subcortical connectivity, in particular in the brainstem and cerebellum, was recently associated with postural impairments in TBI patients (Drijkoningen, Leunissen, et al., 2015), suggesting a possible diffuse pathology across subcortical structures.

Although for most brain areas we found a lower connectivity degree in TBI patients relative to controls, we also found a higher connectivity degree in TBI relative to controls exclusively in the prefrontal cortex. The latter finding, together with the observation that TBI patients have poorer performance in postural control, may suggest that patients have developed a mechanism for stronger cognitive control of such motor actions.

### TBI-Induced Alterations in Functional Networks

Our approach, by focusing on interactions between modules defined by the hierarchical atlas while the brain was at rest, revealed that TBI patients incorporated the prefrontal cortex with a subcortical network. This possibly suggests a mechanism to compensate for TBI-induced subcortical–cortical axonal disruptions, confirmed by the results of our analysis of structural networks, showing decreased white matter connectivity between cortical and subcortical pathways. This disconnection is also consistent with gray matter deficits reported in the frontal and temporal cortices and cingulate gyrus, as well as within subcortical structures including the cerebellum (Gale, Baxter, Roundy, Johnson, 2005; Zappalà et al., 2012).

We also found that TBI patients incorporated the prefrontal cortex with a task-positive network (Fox et al., 2005) employed during the performance of attention-demanding tasks. This suggests more cognitive control and less automatic movement in TBI patients than in control participants.

### TBI-Induced Alterations in Both Structural and Functional Networks and Their Association With Behavior

In many studies of brain networks, changes in functional or in structural network connectivity have often been associated with TBI, yet very few studies have addressed combined effects of changes in both structural and functional connections. Here we have shown that prefrontal brain areas in TBI patients increase their structural and functional connectivity as compared to control participants, and that the resting dynamics of the areas where connectivity increases are negatively correlated with postural control performance. This may refer to a compensatory *plasticity* mechanism that suggests a different mode of balance control—namely, increased controlled processing or less automatic processing of balance movements. Thus, this mode does not represent a successful compensation, whereby increased functional and structural connectivities would lead to increased balance performance, but rather a mandatory change in performance mode that is necessary for accomplishing balance tasks.

Previous work has shown increased prefrontal functional connectivity in patients after TBI (Gooijers et al., 2016; Hillary, Genova, Chiaravalloti, Rypma, DeLuca, 2006; Rasmussen et al., 2008), but as far as we know, we have provided the first evidence that when structural networks are damaged as a result of brain pathology, an associated reorganization of the corresponding functional networks is also established, and vice versa. Moreover, the prefrontal cor tex is the most appropriate locus from which this network reorganization can be orchestrated.

It is well-known that prefrontal areas do not operate in isolation. In particular, it has been widely reported that interactions between the frontal cortex and the basal ganglia play a key role in movement control (Alexander, Crutcher, DeLong, 1990; Aron, 2006; Coxon et al., 2010; Coxon, Van Impe, Wenderoth, Swinnen, 2012; Hikosaka Isoda, 2010; Mink, 1996). Thus, the fronto-striato-thalamic circuit, which enables frontal lobe regions to communicate with the basal ganglia, is involved in a rich spectrum of different functions: motor and oculomotor circuits, executive functions, social behavior, and motivational states (see, e.g., Frank, Scheres, Sherman, 2007). Moreover, there is evidence that the reduced connectivity we have reported in the fronto-striato-thalamic circuit is correlated with reduced subcortical gray matter volume and task performance after TBI (Leunissen et al., 2014a, 2014b).

Furthermore, it has been shown that white matter connectivity and subcortical gray matter volume continue to decrease for up to 4 years postinjury (Eierud et al., 2014; Farbota et al., 2012), which may lead to a reorganization of the prefrontal brain regions to compensate for the damage to the fronto-striato-thalamic circuit. This potential response to the insult is in agreement with our findings and with previous results (Leunissen et al., 2014a, 2014b; Palacios et al., 2013).

### Methodological Issues and Current Limitations

We are aware that the clinical population we studied is small (*N* = 14) and highly heterogeneous, with their time since injury varying from 4 months to 10 years, and their ages ranging from 8 to 19 years. However, patients with moderate to severe TBI in a pediatric population are challenging to recruit. Although we recruited additional patients, these patients had focal brain lesions, and thus their inclusion in our sample would have further increased sample heterogeneity. This motivated us to limit our cohort to *N* = 14 patients, all with diffuse brain injury. Future work, involving larger subject cohorts and/or more homogeneous samples, will be needed to fully address these limitations.

Functional connectivity matrices depend crucially on the specific steps used in the preprocessing pipelines. One step that severely affects connectivity matrices is regressing (or not regressing) out the global signal. Here, in agreement with previous work (Alonso-Montes et al., 2015; Amor et al., 2015; Diez, Bonifazi, et al., 2015; Diez, Erramuzpe, et al., 2015; Mäki-Marttunen et al., 2013; Marinazzo et al., 2014; Stramaglia, Angelini, Cortes, Marinazzo, 2015; Stramaglia et al., 2016), we regressed from each individual time series the global signal, which is well-known to produce more negative correlations in functional connectivity matrices (Murphy, Birn, Handwerker, Jones, Bandettini, 2009; Saad et al., 2012). After we repeated the entire analysis without regressing out the global signal, Figure 3 did not change, but the results for the task-positive network differed from those shown in Figure 4. In particular, the significance of prefrontal regions’ interaction with the task-positive network did not survive correction for multiple comparisons (but did appear in the uncorrected data).

To perform group comparison between the SC matrices, we assessed differences in the module degree statistics, which allowed for localizing brain regions that were *connected* differently in the two groups, but beyond these node-degree group differences, alternative network statistics (based on measures that could go more deeply into the network topology) might identify further group differences (Zalesky, Fornito, Bullmore, 2010). Future work should take this into consideration.

Recent work has suggested that tractography algorithms might produce false-positive connectivity increases in the pathology of TBI (Squarcina, Bertoldo, Ham, Heckemann, Sharp, 2012), mainly due to the existence of smaller fractional anisotropy y values in different tracts after TBI, which ultimately might translate into inaccurate tractography (i.e., counting more streamlines than really exist). We performed a group comparison (FWE, 3dClustSim) of fractional anisotropy values between our groups and found that fractional anisotropy values in prefrontal brain areas were not significant smaller in the TBI group than in controls (results not shown), thus corroborating that the increased connectivity we found in TBI patients as compared to controls was not a consequence of this limitation.

The main motivation to use the brain hierarchical atlas was to examine how our different modules are meaningful for both structure and function. The analysis based on PCA + ICA postprocessing (Figure S1), only used for Figures 3 and 4, is not specific to the brain atlas we calculated, but is based on a functional strategy to extract information beyond the average activity within a region (i.e., the first principal component). Therefore, this strategy will be generally valid for any other brain partition.

## ACKNOWLEDGMENTS

I.D. undertook a 2-month lab rotation to visit the laboratories of S.P.S. and D.M. that was funded by the Health Department of the Basque Government. J.M.C. acknowledges financial support from Ikerbasque: The Basque Foundation for Science; Grant DPI2016-79874-R from the Ministerio Economia, Industria y Competitividad (Spain); and FEDER and Euskampus at UPV/EHU. S.P.S. acknowledges financial support from Bizkaia Talent and the European Commission through COFUND with the research project Brain Aura Mathematical Simulation (BRAhMS; Grant AYD-000-285). S.P.S. was also supported by FWO Vlaanderen (Levenslijn G.A114.11 and G.0708.14), the Research Fund of KU Leuven (C16/15/070), and the Interuniversity Attraction Poles program of the Belgian federal government (Belspo, P7/11).

## AUTHOR CONTRIBUTIONS

D.D., J.G., S.P.S. recruited patients and acquired brain imaging and behavioral data; I.D. preprocessed brain imaging; I.D., J.M.C made the figures; I.D., S.S., P.B., D.M. S.P.S., J.M.C. developed the methods; D.M., S.P.S., J.M.C. designed the research; S.P.S., J.M.C have equal last-author contribution; all the authors wrote the manuscript and agreed in its submission.

## TECHNICAL TERMS

Large-scale functional brain networks: Brain networks obtained by quantifying statistical dependency between the BOLD signals corresponding to different brain regions, built from functional magnetic resonance imaging.
Large-scale structural brain networks: Brain networks obtained by counting the number of streamlines connecting different brain regions, built from diffusion-weighted imaging.
Resting state: A brain condition in which the subject is not asked to perform any goal-oriented task, and therefore, as close as possible to doing nothing.
Default mode network: A brain network that is active when the patient is at rest, composed of parts of prefrontal cortex, posterior cingulate cortex, inferior parietal lobule, lateral temporal cortex, hippocampus, and precuneus. This network is well-known to alter for most brain pathologies.
Postural control deficits: Deficits in balance while trying to maintain a posture against gravity.
Diffuse axonal injury: A traumatic brain injury class characterized by widespread lesions (in comparison to focal lesions) in white matter tracts.
Center-of-pressure trajectory: Spatial trajectory of the application point of the ground reaction force, used in postural control studies.
